# X-ray studies of the transformation from high- to low-density amorphous water

**DOI:** 10.1098/rsta.2018.0164

**Published:** 2019-04-15

**Authors:** Daniel Mariedahl, Fivos Perakis, Alexander Späh, Harshad Pathak, Kyung Hwan Kim, Chris Benmore, Anders Nilsson, Katrin Amann-Winkel

**Affiliations:** 1 Department of Physics, AlbaNova University Center, Stockholm University, 10691 Stockholm, Sweden; 2 X-ray Science Division, Advanced Photon Source, Argonne National Laboratory, Argonne, IL 60439, USA

**Keywords:** ice, water, amorphous ice, phase transition

## Abstract

Here we report about the structural evolution during the conversion from high-density amorphous ices at ambient pressure to the low-density state. Using high-energy X-ray diffraction, we have monitored the transformation by following in reciprocal space the structure factor S_OO_(*Q*) and derived in real space the pair distribution function g_OO_(*r*). Heating equilibrated high-density amorphous ice (eHDA) at a fast rate (4 K min^–1^), the transition to the low-density form occurs very rapidly, while domains of both high- and low-density coexist. On the other hand, the transition in the case of unannealed HDA (uHDA) and very-high-density amorphous ice is more complex and of continuous nature. The direct comparison of eHDA and uHDA indicates that the molecular structure of uHDA contains a larger amount of tetrahedral motives. The different crystallization behaviour of the derived low-density amorphous states is interpreted as emanating from increased tetrahedral coordination present in uHDA.

This article is part of the theme issue ‘The physics and chemistry of ice: scaffolding across scales, from the viability of life to the formation of planets'.

## 1. Introduction

The nature of amorphous ices and their suggested relation to liquid water is the subject of a controversial debate [1–5]. At low temperatures, amorphous ice was found in two forms of different density, namely high-density amorphous ice (HDA) and low-density amorphous ice (LDA) [6], which are connected by a first-order-like transition [1,7–10]. At elevated temperature and pressure a third form, very-high-density amorphous ice (VHDA) [11] was found to exist as a limiting structure. As potential glassy states, the amorphous ices might be seen as frozen liquids of different densities [1,3]. Theoretical considerations, supported by molecular dynamics (MD) simulations, connect the experimental observation of LDA and HDA at low temperatures to the existence of high- and low-density liquid water (HDL/LDL) [12–14]. This two-state model of water [1,2,15] can possibly be used to understand and explain several anomalous properties of water which are critical to a wide range of topics in, e.g. biology, chemistry and geology. Therefore, understanding the structure of amorphous ice as possible immobile water has an important impact [15].

As first discovered by Mishima *et al*. [6], unannealed high-density amorphous ice (uHDA) is made by compression of hexagonal ice at liquid nitrogen temperature. This process was first suggested as high-pressure melting [6] but is now widely accepted to occur at low temperatures due to the mechanical collapse of the crystalline lattice into an amorphous structure [16]. The potential glassy nature of LDA [17] and HDA has therefore been considered controversial [3,18] with others suggesting a more nanocrystalline character [19], a ‘frustrated’ [20] or rather a ‘derailed state along the ice I to ice IV pathway’ [21]. It has been shown that the mechanism of pressure-induced amorphization changes with increasing temperature [20,22]. At elevated pressures (greater than 0.8 GPa) and temperatures above 130 K uHDA transforms further to VHDA [11], potentially entering the ultraviscous (liquid) regime during that step [23,24]. All high-density amorphous ices can be quench-recovered and kept as a metastable state at low temperature and ambient pressure.

While the recovered states of uHDA and VHDA have clearly distinct structures and densities at ambient pressure [25,26], equilibrated HDA (eHDA) [10] or initially called expanded HDA [27], has a similar structure and density compared with uHDA [28]. The densities have been measured using buoyancy by Loerting *et al*. to be 1.15 g cm^−3^ for uHDA, 1.13 g cm^−3^ for eHDA, and 1.26 g cm^–3^ for VHDA [29]. When raising the temperature at ambient pressure, the high-density amorphous ice converts exothermally to the LDA ice [6]. This HDA → LDA transformation has been studied extensively in the literature, using calorimetric measurements [10,30], Raman spectroscopy [8], neutron and X-ray scattering [31–36], nuclear magnetic resonance [37,38] and dielectric spectroscopy [39,40]. It has been shown that different HDA states transform at different temperatures [10,22,27], hence, several sub-states exist within the family of high-density amorphous ice. The limiting state at elevated pressure is VHDA [24], while the most equilibrated and thermally most stable state at low pressures is eHDA [10,27].

Using eHDA as the initial state, recent experiments provided strong indications for eHDA undergoing a glass-liquid transition when heated at ambient pressure above 110 K, prior to the transformation to the low-density state at around 130 K, depending on the heating rate [3,39]. X-ray photon correlation spectroscopy [36] was used to investigate dynamics at the nanoscale, and around the transition temperature of 125–130 K diffusive motion was found, concluding that the transition from eHDA to the low-density phase presumably takes place via a high-density liquid state (HDL) [36].

In Mariedahl *et al*. [41], the structures of the amorphous ices LDA, eHDA and VHDA were studied at 80 K using high-energy X-ray diffraction, and their appropriate oxygen–oxygen pair distribution function g_OO_(*r*) was derived up to *r* = 23 Å. Here we focus on the structural, temperature driven transformation from HDA to LDA ice at ambient pressure. In contrast to previous studies, we compare different initial states (uHDA, eHDA and VHDA) prior to heating as well as different heating rates. By measuring X-ray diffraction over a wide *Q*-range, we obtained high-quality pair distribution functions g_OO_(*r*) during the entire heating process.

## 2. Experimental method

**Figure 1.**
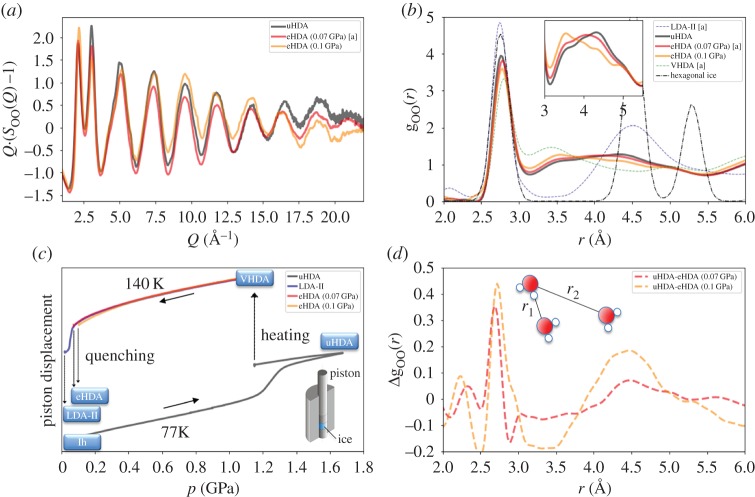
(*a*) Structure factor S_OO_(*Q*) at 80 K shown as *Q*·(S_OO_(*Q*) − 1) for visualizing oscillations up to *r* = 23 Å^−1^. The different lines depict uHDA (black), eHDA decompressed at 140 K to 0.07 GPa (red) and 0.1 GPa (yellow) and quenched at the respective pressure to liquid nitrogen temperature. Panel (*b*) shows corresponding pair distribution function g_OO_(*r*), as well as LDA-II (blue dashed), VHDA (green dashed) and hexagonal ice (black-dash-dot, scaled by 1/3). The inset in (*b*) shows a magnification of the second shell around 4.5 Å. Panel (*d*) shows the difference between the pair distribution of uHDA and the two eHDA states. Panel (*c*) shows the preparation procedure of the different amorphous states, using a piston cylinder set-up (schematic). [a] Data for LDA, eHDA (0.07 GPa), VHDA and Ih have been published in an earlier study [41].

The preparation procedures of the amorphous ices have been described previously in detail by Mariedahl *et al*. [41]. The experiments were conducted at beamline 6 ID-D of the Advanced Photon Source (APS) using identical conditions at a photon energy of 100 keV and analysis procedure as in [41]. Our previous paper [41] describes in detail the experimental parameters and data analysis [42,43]. The partial structure factor S_OO_(*Q*) (figure 1*a*) was obtained from the measured intensity on a 2D-area detector (Perkin-Elmer XRD 1621) and the oxygen–oxygen pair distribution function g_OO_(*r*) was derived (figure 1*b*).

In brief, sample preparation follows the protocol by Mishima *et al*. [6] for making uHDA by compression of hexagonal ice at 77 K, where the amorphous ice is formed above 1 GPa, as can be seen by the pronounced volume change (black curve in figure 1*c*). When warming uHDA at ambient pressure, as later done in the X-ray experiment, LDA-I is formed [44]. Using the pressure device instead, we further follow the protocol by Loerting *et al*. [11] for densifying uHDA to VHDA by pressure-annealing to 160 K at 1.1 GPa. Ice samples have a volume of 300–500 µl, and are compressed inside a steel-cylinder (inner borehole of *d* = 8 mm) using a material testing machine. In figure 1*c*, the different preparation pathways are summarized, and the piston displacement (approx. volume change) as a function of pressure is shown. The LDA-II and eHDA preparation follows the protocol by Winkel *et al*. [45], decompressing VHDA at 140 K to 0.07 (red curve) and 0.1 GPa (yellow curve), resulting in states denoted in the following as eHDA^0.07^ and eHDA^0.1^, respectively. The superscript values describe the pressure at which the samples are cooled rapidly to liquid nitrogen temperature, followed by subsequent decompression at 77 K, which enables recovery of the metastable eHDA states at ambient pressure. Decompression to pressure *p* < 0.02 GPa yields the formation of LDA-II (blue curve) [44]. Note that the pressure device was calibrated using phase transitions at high pressure such as the transformation from hexagonal ice to uHDA. Owing to possible friction effects in different pressure-devices, the precision in the low-pressure region during decompression, in the vicinity of the phase transition HDA-LDA, might have an error of ±0.01 GPa. Therefore, the samples quench-recovered from 0.07 GPa (eHDA^0.07^) show slight differences in their volume (approx. density) compared with studies by Winkel *et al*. [45]. Note, all samples prepared within the same experimental set-up are fully reproducible in their volume/density.

As a sample environment for the XRD measurements, a liquid N_2_-flow cryostat from JANIS was used, capable of operating in vacuum between 80 K and room temperature. The amorphous ice samples were crushed in liquid nitrogen into a powder and subsequently cold-loaded to the Cu-sample holder of the cryostat (see also [41]). The thickness of the powder sample was 2 mm. This procedure of making and transferring the sample gives rise to a macroscopically heterogenous sample, as we will discuss in §4. The area of the sample had a diameter of 5 mm, while the spot size of the unfocused X-ray beam was 0.5 mm, hence 10 times smaller. Consequently, we have measured up to five different positions within the sample, separated by 0.5 mm each.

## 3. Structural difference between eHDA and uHDA

Figure 1 shows the oxygen–oxygen structure factors S_OO_(*Q*) for uHDA (black), eHDA^0.07^ (red) and eHDA^0.1^ (yellow) and the corresponding pair distribution functions g_OO_(*r*). Measurements in S_OO_(*Q*) (figure 1*a*) could resolve oscillations around 1 up to *Q* = 23 Å^−1^, which is emphasized by plotting *Q*·(S_OO_(*Q*) − 1). Figure 1*b* shows that the first coordination shell for the amorphous ices is located around *r*_1_ = 2.75–2.8 Å, which is the nearest oxygen neighbour distance; a detailed discussion can also be found in [41]. The blue dashed line shows the pair distribution of LDA (*r*_1_ = 2.75 Å), which contains the four next neighbours, as shown earlier by integrating the first peak in g_1,OO_(*r*) [26,41,46]. The molecular structure in VHDA (green dashed line) instead contains two additional interstitial molecules between the first and second shell, while HDA has one interstitial molecule, which can be seen by an enhancement towards *r* = 3.6 Å and reduction of the tetrahedral correlations at 4.5 Å. The tetrahedrality of LDA becomes visible by the pronounced second shell at *r*_2_ = 4.5 Å. The ratio *r*_2_/*r*_1_ for LDA and Ih is close to 1.633, the expected value for the tetrahedral O-O-O angle. This was also discussed for the case of liquid water, demonstrating that *r*_2_ (or, respectively, g_2_) is characteristic for the tetrahedrality of the hydrogen bonded network of water [42,47]. In figure 1*b*, clearly the second coordination shell shows the most pronounced difference between VHDA, HDA and LDA. For the three different HDA states the trend is as follows, uHDA (black line) exhibits the highest degree of tetrahedrality at 4.5 Å. For eHDA, the tetrahedrality is most pronounced for the sample that was recovered at the lowest possible pressure prior to the transformation of LDA, namely eHDA^0.07^ (red line). When quench recovering the sample instead at 0.1 GPa (yellow line), the maximum of the second shell in g_OO_(*r*) is shifted toward smaller *r*-values. This trend becomes apparent when directly comparing the difference between g_OO_(*r*)^uHDA^ and g_OO_(*r*)^eHDA^ (figure 1*d*). Pronounced structural differences are found in the first shell around 2.78 Å and in the second shell around 4.5 Å. As reported previously [41], the position of the first peak in g_1,OO_(*r*) is *r* = 2.78 Å for eHDA^0.07^, 2.75 Å for LDA and 2.80 for VHDA, hence the O-O-distance in the first coordination shell increases with the density of the ice [11,41]. This trend of increasing O-O-distance can also be seen between uHDA and eHDA^0.1^. We find the first peak of uHDA at *r*_1_ = 2.77 Å, for both eHDA^0.07^ and eHDA^0.1^ at *r*_1_ = 2.78 Å. According to the literature, eHDA should be expanded [27] towards uHDA, which seems not to be the case here. This conclusion is supported by comparing the position of the first sharp diffraction peak (FSDP) in S_OO_(*Q*) of eHDA^0.07^ at *Q* = 2.14 Å^−1^ in the current study, and at *Q* = 2.0–2.1 Å^−1^ in other studies [10,27,45]. Additionally, the preparation curve shown in figure 1*c* (red line), compared with previous studies by Winkel *et al*. [45], allows us to assume that the density of those eHDA^0.07^ states is different. To resolve this controversy, we propose that the eHDA^0.07^ state used in the present study has slightly higher density than 1.13 g cm^−3^ (as previously reported [28,29]), and is presumably less dense than eHDA^0.1^ but not expanded towards uHDA as suggested by Nelmes *et al*. [27].

## 4. The high- to low-density transition at ambient pressure

**Figure 2.**
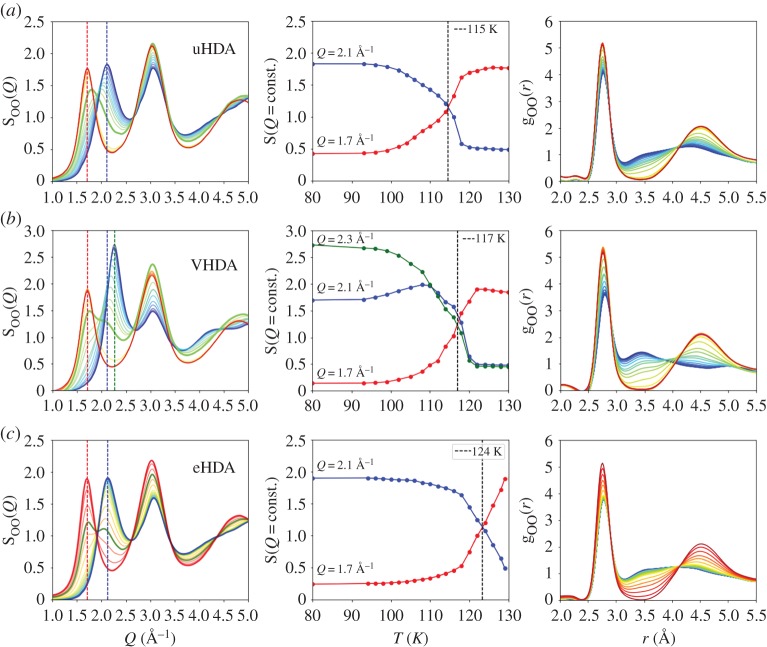
Evolution of S_OO_(*Q*) (left) and g_OO_(*r*) (right) during the high- to low-density conversion for (*a*) uHDA, (*b*) VHDA and (*c*) eHDA (0.07 GPa). Sample warmed from 80 K (blue) to 130 K (red). The overall heating rate is 0.5 K min^−1^, achieved by stepwise heating; each temperature point was measured at up to five different sample positions, the data shown here are taken at the first spot. Column (*b*) shows the intensity of S_OO_(*Q*) at the FSDP position for LDA (1.7 Å^−1^, red), HDA (2.1 Å^−1^, blue) and VHDA (2.3 Å^−1^, green). The S(*Q*) data for uHDA and eHDA have been published in an earlier study as total structure factor instead, as well as the total g(*r*) of eHDA [36].

**Figure 3.**
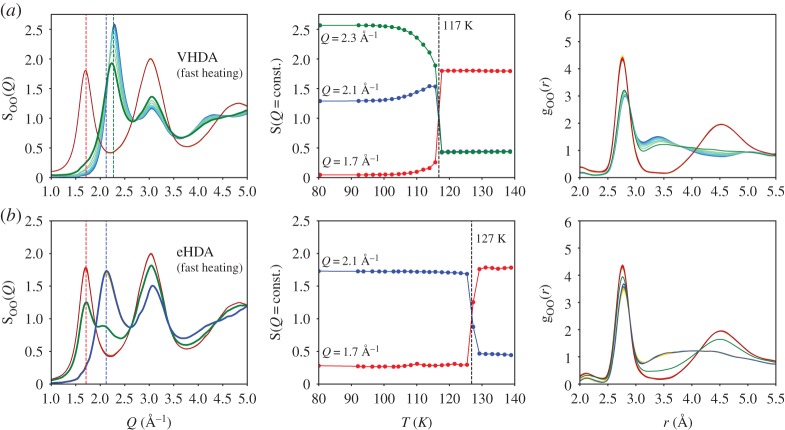
Evolution of S_OO_(*Q*) (left) and g_OO_(*r*) (right) during the high- to low-density conversion for (*a*) VHDA and (*b*) eHDA (0.07 GPa) using a heating rate of 4 K min^−1^. The fast heating took place with a constant ramp of 4 K min^−1^, measuring continuously at the same sample position with an accumulation time of 10 s per point. Samples were warmed from 80 K (blue) to 140 K (red). The middle column shows the intensity in S_OO_(*Q*) at the FSDP position for LDA (1.7 Å^−1^, red), HDA (2.1 Å^−1^, blue) and VHDA (2.3 Å^−1^, green). The total structure factor S(*Q*) of the eHDA conversion was published earlier in the SI of [36].

**Figure 4.**
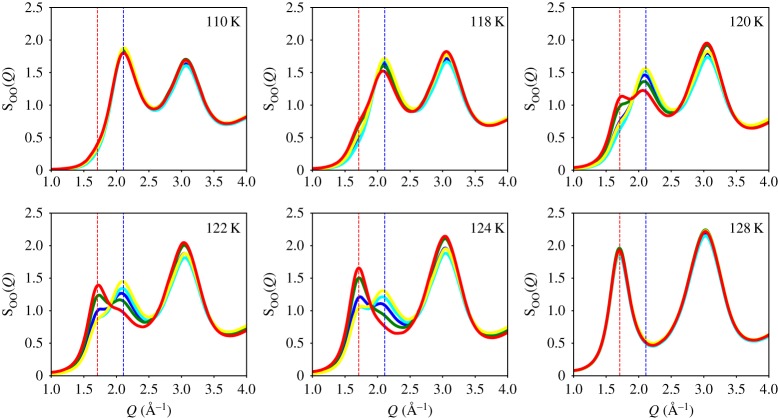
The structure factor S_OO_(*Q*) of eHDA (0.07 GPa) measured at five different sample positions, separated by 0.5 mm, with an unfocused X-ray beam of 0.5 mm diameter. We used a slow heating of eHDA (less than 0.5 K min^−1^); the colour code indicates the different sample positions from 1 (blue), 2 (cyan), 3 (green), 4 (yellow) to 5 (red).

Figures 2 and 3 show the structural transition during the heating process of three different high-density amorphous ices, namely uHDA, VHDA and eHDA. The left column shows the evolution of the structure factor S_OO_(*Q*), all samples have an initial temperature of 80 K (blue lines) and are heated at an average rate of less than 0.5 K min^−1^ to 130 K (figure 2) and 4 K min^−1^ to 140 K (figure 3). The dashed vertical lines mark the position of the FSDP at momentum transfer *Q* = 1.7 Å^−1^ for LDA (red), *Q* ≈ 2.1 Å^−1^ for uHDA/eHDA (blue) and *Q* = 2.3 Å^−1^ for VHDA (green). The S_OO_(*Q*) intensity at those *Q*-values is plotted as a function of temperature in the middle column. We observe the decrease of the HDA peak (blue) and the increase of LDA (red) when heating the samples. As previously discussed [36], and well visible in figure 2, eHDA exhibits the highest thermal stability, whereas uHDA transforms to LDA at 10 K lower temperatures [10,27]. While the eHDA transition develops a double-peak feature within the FSDP, indicating the coexistence of high- and low-density domains within the volume of the sample (thickness = 2 mm) illuminated by the X-ray beam (diameter = 0.5 mm), we find a continuous transition in the case of uHDA, including intermediate positions of the FSDP [36]. It was recently shown [36], that during the discontinuous transition in eHDA the diffusive character of a collective motion at the nanoscale indicated that the transition takes place via a liquid–liquid transition in the ultraviscous regime, at temperatures around 130 K. The dataset showing the total structure factor S(*Q*) in Perakis *et al*. (Figure 2) [36] is identical to the dataset shown here (top and bottom line of figure 2). However, now we calculated oxygen–oxygen S_OO_(*Q*) as well as the pair distribution function g_OO_(*r*) (right column).

Compared with the HDA states, the structure and density of VHDA is distinctly different in accordance with previous work [25,28]. The FSDP in VHDA is at *Q*_1_ = 2.3 Å^−1^ (green line) and shows first a continuous transition to 2.1 Å^−1^ before undergoing a transition to LDA. For all three samples, the second diffraction peak *Q*_2_ = 3.07 Å^−1^ remains at this position during the whole transformation, though a small shift of 0.03 Å^−1^ towards smaller *Q*-values can be observed with increasing temperature.

Figure 3 shows an independent dataset of the two initial phases VHDA and eHDA^0.07^, using a faster heating rate of 4 K min^−1^ in comparison with the data in figure 2. In the estimated heating rate is taken into account, the accumulation time per spot, the step size, number of points measured at the same temperature and dark-images taken of the detector in between the measurements. By comparing the different heating rates and initial phases, one clearly sees that in the case of VHDA and eHDA^0.07^ the transition becomes sharper when using a faster heating rate and is shifted to higher temperatures. We extract as point of transformation the temperature where the red and the blue line in the middle column are crossing and where S_OO_(*Q*) shows either an intermediate position, as in the case of uHDA, or a double peak with almost equal intensity of the two maxima, as in the case of eHDA. The uHDA transition is broad and continuous and takes place at around 115 K. When heating eHDA at 0.5 K min^–1^ the rapid transformation is observed at 124 K, whereas at a rate of 4 K min^–1^ the transformation shifts to 127 K. VHDA, on the other hand, first transforms continuously to an HDA-like state and then nearly discontinuously to the low-density form. The transformation to the low-density state observed here takes place at around 117 K for both heating rates explored (less than 0.5 and 4 K min^−1^). The transition at higher heating rates is clearly sharper while at the slower heating rate the final transition from the HDA-like state towards low-density is very broad (between 113 and 119 K). For the slow heating rate, LDA seems even to nucleate at lower temperatures; whether or not this LDA is formed directly from VHDA or from the HDA-like state cannot be identified here.

Owing to the sample preparation protocol, the powder sample contains macroscopic heterogeneities resulting in different sample positions transforming differently, as shown in figure 4 for eHDA^0.07^. At the lowest temperature (80 K), all measurements taken at different spots show similar S_OO_(*Q*) and identical g_OO_(*r*) [41]. Figure 4 shows the structure factor S_OO_(*Q*) of eHDA^0.07^ measured at five different positions at 110 K (upper left panel) and demonstrate that up to this temperature the heterogeneities do not affect the measured S_OO_(*Q*). When increasing the temperature above this, it becomes evident that the sample exhibits variable transformation temperatures at different spots. This can be due to several reasons. First of all the variable positions have been annealed for different times at the specified temperature, i.e. the measurements at the second spot start when the temperature was already stable for 3 min, whereas the third spot starts after 6 min, and so on. Another possibility can be due to the macroscopic heterogeneities and the loose packing of the grains during the sample loading. This may lead to a variable temperature gradient due to the inhomogeneous thermal contact between sample and sample holder. All the experimental data shown in figures 2 and 3 for the different amorphous states have been measured at spot 1, respectively. After the transition to the low-density state at 128 K (bottom right in figure 4), the five different positions again show identical S_OO_(*Q*). Additionally, we expect the sample after the transformation from HDA to LDA to be more closely packed, because the phase transition is accompanied with a volume change of almost 25%; this is also visible in the measured temperature profile (SI of [41]).

**Figure 5.**
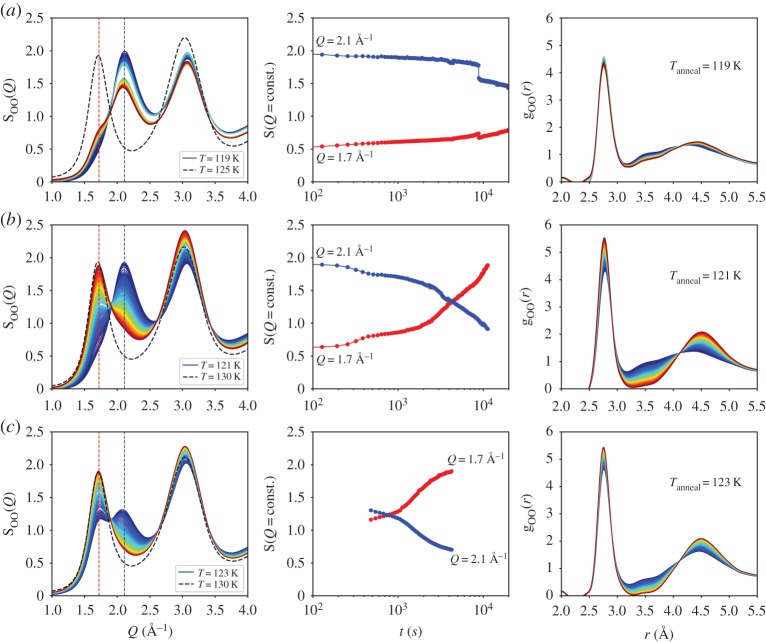
Evolution of S_OO_(*Q*) (left) and g_OO_(*r*) (right) during isothermal heating at (*a*) 119 K, (*b*) 121 K and (*c*) 123 K, using three independent eHDA (0.07 GPa) samples as the initial state. Column (*b*) shows the intensity in S_OO_(*Q*) at the FSDP position for LDA (1.7 Å^−1^, red) and HDA (2.1 Å^−1^, blue); each data point was measured for 60 s.

Furthermore, we investigated the structural evolution in S_OO_(*Q*) as a function of time at constant annealing temperatures. Figure 5 shows eHDA^0.07^ (top row) first heated up to 119 K (dark blue curve) followed by isothermal annealing at 119 K. At this temperature, a small shoulder at *Q* = 1.7 Å^−1^ becomes visible after 2.5 h, indicative of the growing low-density state. For comparison, S_OO_(*Q*) after heating this sample to 125 K (grey dashed line) shows the full transformation. The same evolution for the isothermal annealing can be seen in g_OO_(*r*) (right column), where the second shell at *r* = 4.5 Å becomes more pronounced, indicating that the fraction of tetrahedral motives in the amorphous structure grow while interstitial molecules are displaced. Figure 5 shows also the isothermal annealing at 121 K (middle row) and 123 K (bottom row). When heating the sample first to 121 K, pure eHDA (dark blue) is still visible at *t* = 0. The height of the FSDP in S_OO_(*Q*) at *Q*_1_ = 2.1 and 1.7 Å^−1^, respectively, is plotted as a function of time on a logarithmic scale (middle column). Such transformations as the high- to the low-density transition can be described by an Avrami–Kolmogorov equation, as used and discussed in other studies [31,40]. We have been able to fit the data obtained at 121 K with such a single exponential and obtained a time constant of *τ* = 9810 s (with *α* = 0.9), that is, the transformation still takes place on the timescale of around 2 h. We did not fit the observed stepwise decay, because this could have several possible reasons, but is most probably affected by macroscopic rearrangement of the ice-grains within the 2 mm thick sample. The third eHDA^0.07^ sample was heated up to 123 K at a rate of 0.3 K min^–1^. Owing to experimental issues, it was kept at 123 K for 480 s before we started monitoring the annealing. Thus, it had already partially transformed to the low-density state, as evidenced by a double-peaked S_OO_(*Q*) at the starting time, and we are not able to fit the data in a suitable way. At this temperature, we observe an almost full transformation to the low-density state, within the timescale of 10^4 ^s. For comparison, all three samples were heated afterwards to 130 K (grey dashed lines).

## 5. Low-density amorphous ice and crystallization

**Figure 6.**
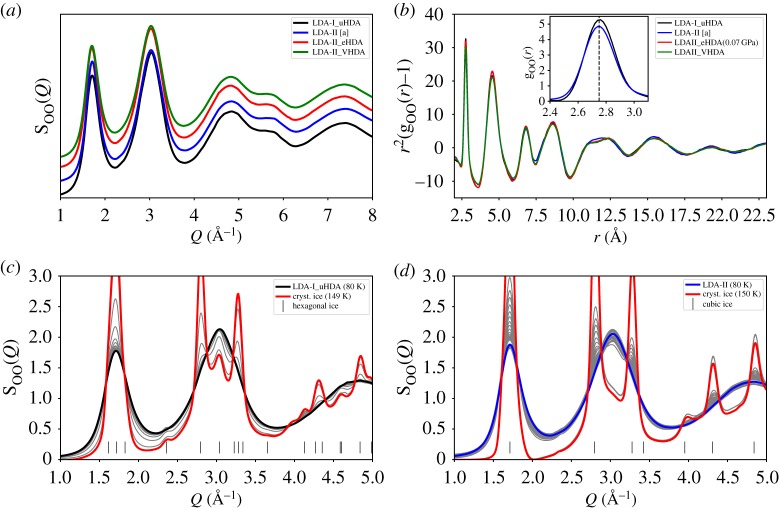
Panel (*a*) shows the structure factor S_OO_(*Q*) at 80 K for differently derived LDA states, (*b*) the corresponding g_OO_(*r*), shown as *r*^2^(g_OO_(*r*) − 1) for visualizing the eight coordination shells up to 23 Å; the inset shows g_OO_(*r*) of the first coordination shell with *r*_1_ = 2.75 Å. LDA-I (black), obtained by warming uHDA inside the cryostat, LDA-II obtained by warming eHDA (red) and VHDA (green), and LDA-II (blue) prepared in the pressure device, as shown in figure 1. Panels (*c*) and (*d*) show the respective crystallization behaviour when warming LDA-I and LDA-II to 150 K.

In the literature, it was already reported that different LDA sub-states exist within the megabasin of LDA. Neutron scattering experiments have shown the structural difference between the so-called LDA-I and LDA-II, as derived from empirical structure refinement [44]. Our X-ray scattering data are consistent with those findings (SI of [41]). The nomenclature mainly differentiates between the initial state of the LDA-sample, namely LDA-I as derived from uHDA and LDA-II as derived from eHDA or VHDA. There are more than two sub-states of LDA [48], but as the differently prepared LDA states show clearly distinct crystallization behaviour it is important to highlight the preparation pathway of the different LDA states. Figure 6 gives an overview of differently prepared LDA states and their different crystallization behaviour. Figure 6*a* shows the structure factor S_OO_(*Q*) at 80 K for LDA-I (black) derived from uHDA inside the cryostat after heating to 130 K (figure 2) and subsequent cooling to 80 K. LDA-II (blue) was prepared inside the pressure cylinder set-up by decompression at 140 K to 0.01 GPa (figure 1*c*). Both LDA-II^eHDA^ (red) and LDA-II^VHDA^ (green) have been derived after *in situ* transformation of eHDA and VHDA; as shown in figure 2, the respective LDA state was cooled from 130 to 80 K. The curves are shifted for clarity by 0.2 each. The comparison of the different S_OO_(*Q*) shows that all four states slightly differ from each other, as also visible in the first and second shell in the pair distribution function g_OO_(*r*) (figure 6*b*).

Figure 6*c,d* shows the structural evolution and different crystallization behaviour when further warming the LDA samples up to 150 K. LDA-I crystallizes (at least partly) directly to hexagonal ice, while LDA-II (both prepared in the pressure cylinder as well as on *in situ* warming, not shown) crystallizes into stacking disordered crystalline ice [49–52], as can be seen by the lack of hexagonal Bragg-peaks at, e.g. *Q* = 2.36 Å^−1^ and 3.04 Å^−1^.

## 6. Discussion

In the current study, we investigated the different structural evolution of high-density amorphous ices when heating at ambient pressure, comparing different initial states, i.e. uHDA, eHDA and VHDA as well as different heating rates. While uHDA transforms continuously to LDA already at 115 K, hence below a possible ultraviscous region, eHDA transforms at higher temperatures (124 K), in a distinct and first-order-like manner. The observed transition starting from eHDA was recently proposed to take place via a liquid–liquid transition (high- to low-density liquid water HDL–LDL), as indicated by measuring a collective, diffusive motion at the nanoscale around the transition temperature of 125–130 K [36]. As previously discussed [36], the double peak in S(*Q*) observed during the transition from eHDA to the low-density state reveals coexistence of high- and low-density domains and is consistent with earlier experiments [8–10]. uHDA and VHDA show a more complex conversion [32]. VHDA first transforms continuously to an HDA-like state but then rather discontinuously to the low-density form at 117 K. This two-step process was reported previously using neutron [53] and X-ray scattering [35]. The HDA-like state transforms apparent first-order-like, similar to eHDA but at lower temperatures.

The thermal stability of eHDA compared with the other states was shown in the literature in many different ways. Although different experimental methods, such as neutron scattering [27,54], calorimetric measurements [10] and dielectric spectroscopy [40] show the same trend, the absolute values differ slightly due to every method using not only a different observable but also different heating rate, due to experimental restrictions. In the current study, we now compared different heating rates within the same experimental method and found that when using a 10× faster heating rate of 4 K min^−1^, the conversion appears distinct both for the HDA-like state starting from VHDA and eHDA^0.07^ (figure 3). Remarkably, the structure factor S_OO_(*Q*) and the pair distribution function g_OO_(*r*) of eHDA^0.07^ remain almost constant while heating from 80 to 126 K, hence prior to the transition.

Isothermal annealing experiments at temperatures below the transition temperature show that the structure of eHDA^0.07^ is stable at 119 K for almost 2 h, while at 121 K it starts transforming within the timescale of 1 h, and at 123 K within 480 s. These results are consistent with isothermal dielectric measurements [40] investigating the transformation kinetics of eHDA. For 123 K, the dielectric measurements [40] derive a timescale of 10^4^ s for the conversion, and a slightly larger value for 121 K. An exponential fit to the X-ray data calculated a time constant of *τ* = 9810 s and is therefore consistent with the dielectric measurements [40]. The timescales at the other temperatures are difficult to compare, because here measurements at times longer than *t* > 10^4^ s were not conducted, and both eHDA samples at 119 and 121 K did not fully transform to LDA within that time frame. Slight deviations can have different reasons, as thermal contact or simply the fact that all X-ray measurements on these samples have been done at the same sample position. Additionally, we demonstrated that these powder-samples exhibit macroscopic heterogeneities and different points within the sample can transform on a slightly different timescale.

The different crystallization behaviour of LDA-I and LDA-II was investigated previously in the literature. Neutron scattering experiments [44] indicated a higher thermal stability of LDA-II compared with LDA-I and calorimetric measurements show small differences in the enthalpy of crystallization. The X-ray diffraction data, presented here, show the same crystallization behaviour of LDA as previously discussed by Seidl *et al*. [55] as parallel versus single crystallization kinetics. That is, LDA-I derived from uHDA crystalizes into hexagonal ice, whereas LDA-II derived from eHDA crystallizes to stacking disordered ice (or also commonly denoted cubic ice) [49–52], and they concluded that uHDA contains crystalline hexagonal remnants [55]. Recent MD simulations [56] of both uHDA and eHDA at high pressures showed a ‘lack of polydisperse ice-like structures', but a weak indication that uHDA contains a ‘non-negligible number of Ih-like molecules’ at elevated pressures of 1.4 GPa, clusters composed of 5–10 molecules [56]. We do not find direct evidence for uHDA containing crystalline hexagonal ice, because e.g. g_OO_(*r*) of uHDA in figure 1 does not show a feature at *r* = 5.3 Å, indicating that there are no significant numbers of distinct hexagons present. A small shoulder might be present at *r* = 5 Å, which could be interpreted as distorted hexagons, but cannot be identified free of doubt. On the other hand, the direct comparison of the pair distribution function between uHDA and eHDA (figure 1) shows an enhancement of g_OO_(*r*)^uHDA^ around r = 4.5 Å compared with g_OO_(*r*)^eHDA^, indicating that uHDA contains an increased amount of tetrahedrality within the molecular structure. The enhancement at 4.5 Å can be associated with the ordering of two adjacent tetrahedra, which finally leads to the different crystallization behaviour. The double peak feature in S_OO_(*Q*)^eHDA^, and its absence in the case of uHDA, are further indications for the existence of different sizes of either domains of differently arranged tetrahedral configurations or nanocrystalline remnants or amorphous domains of different density.

## 7. Conclusion

X-ray diffraction experiments support earlier suggestions that unannealed high-density amorphous ice (uHDA) is more related to a collapsed crystalline state, while equilibrated high-density amorphous ice (eHDA) is a glassy state [36,39]. This conclusion is backed by three experimental observations: (i) while not seeing direct evidence for crystalline Bragg-peaks in the reciprocal space, the pair distribution g_OO_(*r*) of uHDA contains an enhanced amount of tetrahedral correlations at *r* = 4.5 Å compared with eHDA; (ii) the distinct structural evolution in reciprocal space supports the view of a first-order-like transition from eHDA to the low-density state, where a double-peaked S_OO_(*Q*) reveals the coexistence of high- and low-density domains; uHDA instead lacks this double peak feature and transforms continuously, indicating the existence of much smaller domains; and (iii) LDA-I and LDA-II, derived from uHDA and eHDA, respectively, show different crystallization behaviour, with LDA-I crystallizing into hexagonal ice. Similarly, when using VHDA as an initial state, a continuous transition to a HDA-like state is observed, followed by a rapid transition to LDA. Both the structural evolution of this HDA-like state and the crystallization behaviour of the derived LDA-II indicate that this derived HDA state is closer to eHDA, hence of glassy nature as well.

## Data accessibility

The datasets supporting this article have been uploaded to the repository, available from https://github.com/Katrin-Amann-Winkel/Amorphous-ice-XRD-data.
